# Supplementary material for: Illness Narrative Master Plots Following Musculoskeletal Trauma and How They Change over Time, a Secondary Analysis of Data
Source: Behav Sci (Basel). 2024 Nov 19;14(11):1112. doi: 10.3390/bs14111112 (PMC11591027; doi:10.3390/bs14111112)
Supplement: Supplementary file 1 [file behavsci-14-01112-s001.zip › behavsci-3161564-supplementary.pdf]

<u>**Supplementary File**</u>

Examples of the 5 Stage Categorical Form Analysis

The below are examples of the stages of analysis undertaken.

Stage 1 of the Categorical Form Analysis

This stage required the research to consider quotes and link the quotes to each participant

Narrative	Quote	Factors that influence th	Narrative	quote	notes	Narrative	quote	notes	Narrati	Quote	Notes	Narrati	Quote	Note	Narrative	quote	notes	Narrative pivot points	ence that change the view of	Notes	Dominant narrative
	everything that I could to stop myself from going into the back of the car. That didn't end up working. So instinctively I had then moved across onto the other lane of the traffic"..."So it was in a million pieces. After that I came too, on the ground. I was unconscious for a little bit after the accident"..."I remember them saying that my foot was quite messed up. I don't know exactly if it said that there was a compound fracture, but near to a compound fracture "..."After I'd had			done, which was amazing. And within the next day after having all of this done, they'd had blockers put in to reduce the pain...but then they were getting me to walk on my femur (laughs), after I'd just had the operation, which I thought was amazing. " ...And then from there, same thing again. Came out of surgery, felt great, had the block around my			give me an idea of things you're struggling with? Respondent:Walking! Getting up. Doing anything. I need support , I need... I've got an A-frame that they've given me and also a set of crutches. I'm trying to use... I'd used the A-frame the whole time that I was in the hospital, just to go to the toilet, with assistance initially, as you would. And then by the end of it I was becoming self-sufficient ,			We've talked about the physical side of it, how about the mental side of it, how's things like your concentration and things like that, since the injury? Respondent:Initially terrible. I was noticing when I was writing a text message or anything like that, I couldn't write a sentence. I couldn't write			'recovery' I go, okay, if I can't do that thing, I'm going to do something else. If I think my legs... well I'll try and do something with my upper body,		Quest automythology				story begins with a description of the accident which was very serious and introduces a potential tragic master plot. Significant injuries are		

Baseline | 6 months | 12 monthsa | Demographics & information

Stage 2 of the Categorical Form Analysis

The next stage organised each participants stories for each time point

B2

The story begins with a description of the accident which was very serious and introduces a potential tragic m
needed and blood transfusions. The restitution master plot was expressed by the idea of going back to normal
following the accident he thought it would be short but had to revise that he states "a couple of weeks and I'll b
I'm... if I can walk before Christmas I'll be laughing." The idea of the change in time scale is not considered a pr
expected, but will still occur. Challenges were identified including the need for further operations and conside
considered limiting. A sad narrative is expressed identifying what is possible now currently versus what he use
anything that I was used to, going to the toilet, shower, drive, ride a motorbike obviously (laughs). Anything like

A	B	C	D	E	F
**Participant**	Baseline	6 months	12 months	Comments around narratives	Comments on time
	The story begins with a description of the accident which was very serious and introduces a potential tragic master plot. Significant injuries are identified, multiple surgeries needed and blood transfusions. The restitution master plot was expressed by the idea of going back to normal and that was combined with revised times scales, initially following the accident he thought it would be short but had to revise that he states "a couple of weeks and I'll be back to normal. And now it's gone from a couple of weeks to I'm… if I can walk before Christmas I'll be laughing." The idea of the change in time scale is not considered a problem, because restoration is going to take a bit longer than expected, but will still occur. Challenges were identified including the need for further operations and considerations around the experience of pain are mentioned but not considered limiting. A sad narrative is expressed identifying what is possible now currently versus what he used to be able to do and stopped his plans he states "I can't do anything that I was used to, going to the toilet, shower, drive, ride a motorbike obviously (laughs). Anything like that I can't do, anything, bedbound pretty well….all that normal walking and everything like that, I can't do". The didactic narrative was identified at the hospital when something was made	The didatic narrative was expressed whilst considering the learning from experiences which affected the view forward this included experineces around surgery e.g., the interview starts by identifying recent procedures and advice given by health care progressionals "I had wires taken out of my foot", I had an x-ray…they said they were going to 3D print a bone….but then after I had a CT scan….they've said that because of all the bones that have broken it is not going to happen..They've pretty well told me to go and live my life and if it hurts come back". This is immediately followed by a statement around the quest narrative which says "I've turned it into a bit of a postive and really just cracked on. I have started to live my life a bit more". Clear identificaiton of what can be done is made e.g., An action/heroic narrative is used to create change in the present to meet short term goals which buy into the restoration plan. For instance the idea of small goals and small steps provide a way to make progress. For instance he states "I'm able to drive a car, I can ride a motorbike. Yeah, I'm back to normal for that sort of stuff . I suppose the big thing that I'm wanting to, the next step for me would be to start going to the gym. I'm going to start going to the pool, start swimming, which	The story begins with a focus on progress and a holiday to Australia where they were not using crutches but now is, which was identified as a 'set back' and wanting to be without them, this places some uncertainty around the abilty to be restored, he identifies known limits of being able to walk around limits of walking in one go at around a mile, but witih more walking he says that he would 'feel it [pain] quite a lot'. Part of the problem is related to how he walks for instance he states walking 'on the ball on my heel' which creates numbness and difficulties moving up and down stairs are identified. Problems are identified as they state "Recovery, yeah. Day by day it feels a bit different. My left foot's still giving me grief and my right knee is still quite painful. So, I do struggle with walking from time to time, which is essentially, yeah, what I want to be able to achieve.". Achievements and improvement through repating tasks are identified as current gains including, sitting up and down, standing up, using the toilet, putting trousers on. But no recent physitoherapy contact has been made due to the physiotheapist 'pushing back [the time]' each time it is arranged. A didatic narrative aroud a past knee reconstrcution and rehabiliation is idetifnied as a way to approach not having seen a physiotherapist as he states 'i am keeping in my what i need to do…working on posture and walking and things like that'.		Questions: is the future known or unknown? What about it is known? What about it is unknown? What about the future is wanted? What about the future is accepted? What about

A summary of these comments were also made by time for instance.

Participant 1 notes

Time 1

The shock and reaction to the accident

Participants were able to recall the events in a vivid way following an accident. Each event resulted in significant injuries, multiple surgeries and often blood transfusions. P15 states “"my left foot was completely smashed. It was literally gone...I just remember seeing my boot isn’t on there.". Some focused initially on the difficulties and challenges. For instance, a state of shock at the initial events is mentioned by P4 and P12 stating “I remember feeling strongly like, “this can’t be happening”. P12 wondered if he was dead and asked a bystander the question. P4 describes being taken to a hospital away from his home, he locates this experience by stating “I didn’t know anyone, there were no visitors allowed, I have zero connection to [name of city]”. He identifies this time as “not being in a good place mentally” and “feeling very alienated” and not motivated to do anything. P24 identifies the accident as a head on collision and being taken to hospital, She identifies confusion about the situation and not knowing what was going on, she says people who were there identified her as being in an out of consciousness, only after a while did she realise that she couldn’t move her legs, and she states “I wasn’t feeling a lot of pain”. P24, initially focuses on wanting to get out of hospital and how that was best achieved.

Some participants identified acceptance and understanding of the situation initially. An instant sense of acceptance was illustrated by P10 stated “I'm quite a control freak, so letting someone do what they've got to do, comes hard. But I get to the point where there's no choice, and you've got to go with what you're being told is right". P17 identified experiences of accidents before whilst mountain biking, the nature of his injuries slowly became understood when he tried to get back to his car after the crash. He states “I was using the bike as a crutch, just to try to get out...And I said to [name of friend], “I can’t go any further.” So, he said, “Ambulance?” and I said, “Yes". For P14 previous experiences provide insight and help acceptance. He identifies having epilepsy and the resulting blackouts injuries. His response to the initial experience was "Not again. (Laughter) Yeah. Here we go again....I was not frightened at all. I know what to expect." He identifies that he understands what he can do that his body ‘tells’ him how to engage with injuries and is able also to joke about the experience stating "running the London Marathon this year is definitely not in". P8 identifies the significance of the injury, but the importance of accepting it and getting on with it. His reaction following surgery illustrates this stating "that morning and the day of surgery, I was looking forward to the next one to get things done. ". Almost instantly, he identifies the losses which will not be possible again like riding his motorbike or quadbike. Within the hospital he recognised the implications "I understand completely that not everybody can have a full recovery".

Some participants focused more on the implications or impact early on. For P15 the accident represented a bigger impact on this life, as it occurred whilst on a motorcycle attending the one of two new jobs, which would provide him with full time work. This followed a time of being unemployed and living off savings. The instant implications were stated, "this accident's kind of just cancelled all that. They're both roles that would have needed me… required to be on my feet constantly. And I've had a few physios saying that I'm not going to be able to walk for the next, like, six months to maybe a year properly”. P7 identified an escape type narrative to the hospital experience, the heroic narrative early in the hospital, stating a desire to get out of the hospital, that became a goal for him, 'not to get comfy' in the hospital, this contrasted a view on others who seemed comfortable to 'do their bedpans and watch all their TV'.

Several participants identify the significant and overwhelming experiences of pain. The experiences of pain are expressed around surgery, involving having to put up with spasming muscles and 'horrible pain' and waiting on medication in hospital to provide relief for the pain and the urgent need for it before it arrived. P18 identifies details pain experiences during the transfer to hospital, stating "I had to get my mum to tell me every time there was a bend...Every time I tensed my leg, it was so painful. It was just another type of pain; it was horrible." P20 identifies the seriousness of it with broken bones in a short way, the pain was identified but again described objectively and briefly. For instance, he states "Initially they give you a lot of morphine to help with the pain and then gradually after about five or six days they took the lines off me and just gave me morphine orally, if required." and later states initially it was pain like he had 'never experienced before".

Stage 3 & 4 of the Categorical Form Analysis

This stage of the analysis required the lead author to bring together ideas for the narrative master plots. This was undertaken in different ways including by narratives and by time

Analysis by Narratives

Initial early reactions and the birth of stories

The recollection of the moments when experiencing the accidents and injuries and the moments that followed provided the starting point for identifying meaning in relationship to time

Reactions at the point of the accident

Participants often started the interviews by describing the accident. The description of the events was represented by a disembodied narrative describing what happened in detail and clearly articulated. At the point of the accident there was a sense of shock. For instance, P12 stated “I remember feeling strongly like, “this can’t be happening”. P15 could not see his motorcycle boot on his foot and that his foot was dangling off his leg. P4 was initially taken to a hospital away from his home. He locates this experience by stating “I didn’t know anyone, there were no visitors allowed, I have zero connection to [name of city]”. P24 identifies being in a state of confusion about what was happening, being told afterwards by others that she was slipping in and out of consciousness.

Several participants identify the significant and overwhelming experiences of pain. The experiences of pain are expressed around surgery, involving having to put up with spasming muscles and 'horrible pain' and waiting on medication in hospital to provide relief for the pain and the urgent need for it before it arrived. P18 identifies detailed pain experiences during the transfer to hospital, stating "I had to get my mum to tell me every time there was a bend...Every time I tensed my leg, it was so painful. It was just another type of pain; it was horrible." P20 identifies the seriousness of the accident with broken bones in a short way, the pain was identified but again described objectively and briefly. For instance, he states "Initially they give you a lot of morphine to help with the pain and then gradually after about five or six days they took the lines off me and just gave me morphine orally, if required." and later states initially it was pain like he had “never experienced before".

Moments that impact the stories and future

Specific moments of change or accomplishment early on during rehabilitation were often mentioned which appeared to provide a seed of hope for participants. P10 and P17 identifies the need for achieving small changes and that acted as P17 states as ‘something positive’. P1 P17, P18 identify specific moments of change, this included taking first steps standing up right for P17, and for P1 it was walking, he states “I didn’t think I was going to be able to and then all of a sudden, within two minutes I was walking” (P1). P18 talks about identifying a turning point, he states “did my stairs, and then got a wheelchair back. So that was...Thursday was my turning point; my good day".

Other moments that impacted the participant adjustment and change their reality were also mentioned. This included not being able to complete a task which they thought could be possible, and recognition of a limit their body had or understanding physical limits or being forced to stop in the present. For instance, P1 identified not being able to navigate stairs and the act of trying made him realise it is not possible. P18 identified low energy levels and limited allowance of movement for instance he states “I wasn’t allowed to do any more weight bearing than just toe touch weight bearing”.

Awaiting an assessment, result or change in the future was identified as a moment which meant life and the future was on hold e.g., P15 identified “they [HCPs] made me aware that a year down the line things can still go wrong with the grafting”. P17 recognised that there could be ‘something’

wrong identified during recovery which, will, in the future prevent him doing his job, he identified this within a context of having a lot of recovery identifies an understanding of order of which mends and change will occur.

For some, because of the injuries experienced and limitations placed on them the future is known and it may not be possible to change, to access previously identified hopes from rehabilitation or even want to engage with. This could be expressed in a contrast of what is possible in the past is not possible now. P1 identifies that he cannot do what I used to do “I can’t do anything that I was used to, going to the toilet, shower, drive, ride a motorbike obviously (laughs). Anything like that I can’t do, anything, bedbound” (P1).

P4 acknowledged the physical limitations current and what they mean in within his present situation. He states “Well, it really is just the physical limitation, right? Like, I cannot walk down the block; like, I cannot imagine getting on a plane and walking through an airport. Do you know what I mean? Like, carrying a whatever. I just cannot; nowhere close to that at the moment”. P12 identified the limited expressed with getting dressed and limitations in movement currently. Following the accident P17 identified that he would be more risk adverse which could narrow what activities would be engaged with and impact the future..

A need for action narrative

An action or need for action narrative appeared to be prevalent across participants. The basic plot was that if I can engage in rehabilitation right now in the present then a better future is possible. Identified in another way there is a need to act now to create change and engage in a challenge then I can reach my goals and progress, meaning change and hope is possible. The need for action was partly linked to staying motivated during rehabilitation as P4 states “I need to do exercises and do that and really stay motivated. So, I think just staying positive and, like, focusing on, like, the short-term day-to-day, the exercises, I think that’s helpful. because they know that people easily get demotivated”. The importance of accomplishment and motivation is identified by P1 who states “if you don’t see everything as a challenge and if you don’t try to accomplish it, it’s not going to be worth it. And if you’re sitting there going, my recovery, dwelling on it or anything else like that, then you’re not going to get through it as quick as what you want to .” This narrative had heroic elements to it but placed emphasis the need for motivation narrative was represented more as a challenge identified for the future and need for action now, rather than a challenge met in the past (heroic narrative). Statements representing this included ““I’m definitely going to be able to keep going and keep moving and keep going forward with this.” (P1), "I’ve just got to get better, you know, determined that I’m going to push through it." (P12) and "if you have that positive mindset, you're going to physically do whatever you can do to make yourself better" illustrating a heroic outlook.” (P15). P24 illustrates action in overcoming challenges for instance including this “I have been back in to see the consultant this week, on Tuesday, but again, I had to do that myself and get myself in the car. We had to drag me into the car and drive me to [hospital location]”

P12 identifies a need around independence, he states “to be independent, I’ve got to put the work in". He combines a future want or hope with the need for action now. Two participants P7 and P24 had a very specific need of getting out of hospital as soon as possible. P24 arranged home adjustments whilst in hospital and, at the same time, engaged with health care professionals in a directive way. She stated “every time I saw an OT, I flagged them down until I found someone that was willing to work with me”. P7 identified a goal of ‘not to get comfy' in the hospital, this contrasted a view of others in the hospital who seemed comfortable to “do their bedpans and watch all their TV”.

Wanting or needing to look forward and accept; the birth of the quest

The ability to look forward, to recognise what had happened and accepting the impact of that had happened in what seemed like an instant way for some. For instance, P10 identifies having acceptance to what he was being told without choice. P14 and P17 identify past experiences and injuries which influence acceptance. For instance P14 identifies accidences from epilepsy he states “Not again. (Laughter) Yeah. Here we go again....I was not frightened at all. I know what to expect." A slightly different view of acceptance and embracement of change was represented by P14 who states “other people might think of me as disabled. I think I’m extremely able. Yeah. It’s a matter of attitude, isn’t it? " He goes on to illustrate an accepting nature of his attitude that makes everything useful or useable, akin to an adventure narrative. He considers how he embraces difficulties or challenges is stated "*Healing, physical healing takes longer when I’m just there depressed.... And, of course, having a good time is much more enjoyable. Yeah. There’s always a silver lining. And if you can't find one, you can always create one....It’s always been the same. I always was of the opinion, well, if life gives you lemons, make lemonade*".

Wanting to move past the present into a better situation or future was identified by several participants. P8 identifies the value of surgery for moving things forward He states “"that morning and the day of surgery, I was looking forward to the next one to get things done". P1 illustrates the importance of working with what is possible he states “I always just try and think about the positive of it. If I’ve hurt my knee, then I’ll think okay, now I can do some more stuff with my upper body and just keep that in mind, in a physical sense, that’s the way I sort of look at it”. (P1). P12 identifies the importance of getting around problems he states "I’m doing okay. I’m quite determined to get round it. And I can get about on my crutches quite good, in fact, I feel sometimes that I don’t need these crutches".

P20 and P18 identify the importance of changing as a result of what has happened. P20 states “" I just feel lucky to be alive you know and its completely changed my life, my attitude, you know I just want to look after myself more. I’ve lost, my weight is down, I’ve never been so... my weight has never been so low. ". P18 identifies a transformation and quest auto mythology as part of his narrative because of the change that has occurred he states “he has to 'learn to live with the injuries', 'good clean living, eating healthily....get as mush rest and stick to their [health care professionals]

advice" P24 identifies marking off what may not be possible in terms of function and the future. She states "I am aware that that is maybe not possible, but I'm trying to sort of keep quite a neutral mindset, not worry one way or the other because I don't think that that's helpful"

The future and establishing what is normal; the birth of the restitution

The restitution narrative and returning to normal. Normal could be being like new (P4) as in a being fixed, or normal could refer to activities or roles for instance For P10 being with his children is important and part of what he wants to return. For P10 restoration towards being mobile is needed as he states "as long as I can get out with my kids, you know...just stuff we did before , that's all really." P14 places restoration in steps states "once I've achieved one [goal], then I can do the next one…Do the same as before, a little bit faster".

How the future was viewed was different across individuals. For some the future restoration was linked to healing times, something which was known. P17 states the "hip eight weeks, the ribs might be slightly quicker". P20 expressing a similar view but within a timescales when considering recovery "He states "I'm just sort of slowly recovering at home now but it's going to be four to six months before I fully recover I think, or could be longer." P18 supports identifies that knowledge of recovery is evident from past injuries of the shoulder which required effort for restoral, this can be considered by the statement "So I'm thinking my leg will be the same [recovery and restoral], once I get through it. Like I said, little wins make a big, big difference at the end of the day." . However, this could require adjusted time scales or identification of a known outcome but unknown date or a more generic outcome in the future. An example of adjusted outcomes is identified by P1 who states: "a couple of weeks and I'll be back to normal. And now it's gone from a couple of weeks to I'm... if I can walk before Christmas I'll be laughing." (P1) P12 talks about the possibility of going back to work a job which is enjoyed, but there is an element of being unsure if that will happen and identification that it depends on progress with his arm and walking which is unknown. A more open future without specific dates was identified by P4. P4 leaves more of an open door as to when normal will happen stating "I just know it's going to take a while, but, like, I'll be back good as new at some point".

P10 acknowledged his past identity as being athletic and physically able. He acknowledged this identity as a loss, contrasting the past with now for instance "I was doing the dead lift, 150kg, you know, one or two, and then you'd like pass out...I think my personal record is 350kg on a leg press...And to see my legs now, I've lost so much muscle mass.". This was followed up by identifying what cannot be done currently, one aspect was having no strength to move his leg inward. These experiences affect him as he states 'that's hurting me, nor hurting physically, that's hurting emotionally at the moment….literally got nothing there, but I don't know how to do anything about that"

Analysis by time

Baseline time point zero

Time point 1: Reactions at the point of the accident and disembodied descriptions

The disembodied narrative was consistently used to describe the events of the accident. The basic plot is that of a tragic accident which is identified in details and reactions at the time as they look back. Participants often started the interviews by describing the accident. The description of the events was represented by a disembodied narrative describing what happened in detail and clearly articulated. At the point of the accident there was a sense of shock. For instance, P12 stated “I remember feeling strongly like, “this can’t be happening”. P15 could not see his motorcycle boot on his foot and that his foot was dangling off his leg. P4 was initially taken to a hospital away from his home. He locates this experience by stating “I didn’t know anyone, there were no visitors allowed, I have zero connection to [name of city]”. P24 identifies being in a state of confusion about what was happening, being told afterwards by others that she was slipping in and out of consciousness.

Several participants identify the significant and overwhelming experiences of pain. The experiences of pain are expressed around surgery, involving having to put up with spasming muscles and 'horrible pain' and waiting on medication in hospital to provide relief for the pain and the urgent need for it before it arrived. P18 identifies detailed pain experiences during the transfer to hospital, stating "I had to get my mum to tell me every time there was a bend…Every time I tensed my leg, it was so painful. It was just another type of pain; it was horrible." P20 identifies the seriousness of the accident with broken bones in a short way, the pain was identified but again described objectively and briefly. For instance, he states "Initially they give you a lot of morphine to help with the pain and then gradually after about five or six days they took the lines off me and just gave me morphine orally, if required." and later states initially it was pain like he had “never experienced before".

Master plot 1, time point 1: Negotiating the present and a need for action narrative

An action or need for action narrative appeared to be prevalent across participants. The basic plot was that if I can engage in rehabilitation right now, or I act in the present then a more independent future or, future with better outcomes is possible. Key components of the narrative appeared as having a focus on making small gains or taking small steps to improve and this is to guard against being demotivated which was recognised as a real possibility. This narrative was created in a context when past progress has not been undertaken, and the certainty of future outcomes and progress is limited. For instance, P4 states “*I need to do exercises and do that and really stay motivated. So, I think just staying positive and, like, focusing on, like, the short-term day-to-day, the exercises, I think that’s helpful. Because…people easily get demotivated*”. A core objective of the narrative was being able to take action that could allow access to potential change efficiently. For some this was contrasted against an idea of dwelling on the problem or the past which could prevent it. P1 and P12 identified the importance of an intention to change, or a need or commitment to change. For instance, P12 states "*I’ve just got to get better, you know, determined that I’m going to push through it."* and later he says “*to be independent, I’ve got to put the work in*". P1 states “*if you don’t see everything as a challenge and if you don’t try to accomplish it, it’s not going to be worth it. And if you’re sitting there going, my recovery, dwelling on it or anything else like that, then you’re not going to get through it as quick as what you want to*”. Other components of this narrative included an idea of a positive mindset and keeping motivated, P15 states "*if you have that positive mindset, you're going to physically do whatever you can do to make yourself better*" (P15). Another

aspect was looking forward to the next step or change. P8 identifies the value of surgery for moving things forward He states “"*that morning and the day of surgery, I was looking forward to the next one to get things done*". This narrative may also be supplemented by a need and strong motivation to accomplish a specific task or goal. For instance, two participants P7 and P24 had a very specific intension of getting out of hospital as soon as possible. P24 arranged home adjustments whilst in hospital and, at the same time, engaged with health care professionals in a directive way. She stated “*every time I saw an OT, I flagged them down until I found someone that was willing to work with me*”. P7 identified a goal of “*not to get comfy*” in the hospital, this contrasted a view of others in the hospital who were perceived as comfortable to “*do their bedpans and watch all their TV*”.

The narrative was supported by moments of overcoming challenges. For instance, P24 illustrates action in overcoming challenges stating “*I have been back in to see the consultant this week, on Tuesday, but again, I had to do that myself and get myself in the car. We had to drag me into the car and drive me to [hospital location]*”. This narrative was helped by specific moments of change or accomplishment early on during rehabilitation. P10 and P17 identified the need for achieving small changes which provided ‘*something positive’*. P1, P17 and P18 identify specific moments of change, this included taking first steps standing up right for P17, and for P1 it was walking, he states “*I didn’t think I was going to be able to and then all of a sudden, within two minutes I was walking*” (P1). P18 talks about identifying a turning point, stating “*[I] did my stairs, and then got a wheelchair back. So that was...Thursday was my turning point; my good day*". P14 identified the importance of steps and states "*once I've achieved one [goal], then I can do the next one...Do the same as before, a little bit faster*".

Attempting movement and rehabilitation could also identify limits of action and possibilities in the future. This experience demonstrated that the process of change was not linear and this could act to limit and challenge this narrative master plot. For instance, P1 identified not being able to navigate stairs and the act of trying made him realise it is not possible. P18 identified low energy levels and limited allowance of movement stating “*I wasn’t allowed to do any more weight bearing than just toe touch weight bearing*”. Other aspects which challenged the idea of action occurred if participants were awaiting an assessment result or for change that could happen in the future. This could mean that life and the future was on hold. For instance, P4 acknowledged the physical limitations current and what they mean in within his present situation. He states “*Well, it really is just the physical limitation, right? Like, I cannot walk down the block; like, I cannot imagine getting on a plane and walking through an airport. Do you know what I mean? Like, carrying a whatever. I just cannot; nowhere close to that at the moment*”. Other examples provided by P1 and P12 identify difficulty with day-to-day tasks such as taking a shower, going to the toilet, getting dressed and other limitations in movement or activities. Finally, following the accident P17 identified that he would be more risk adverse which could narrow what activities would be engaged with and impact the future.

Master plot 2, time point 1: The future and establishing what is normal; the birth of the restitution

The focus of restitution and basic plot was that I will return to some form of ‘normal’. The reference of normal was focused on activities and relationships, roles or social identities previously assumed. The way the narrative changed was around what was represented as ‘normal’ for each participant. Normal was represented in the following ways: (a) normal as being ‘like new’ (P4) as in a being fixed, or, (b) normal could refer to activities, specific movements,

function or specific roles. For instance, P10 identifies a need for restoration towards being mobile for his children, he states "*as long as I can get out with my kids, you know…just stuff we did before , that's all really*." The importance and realisation that normal could not be a straight fix is illustrated by P10. P10 acknowledged his past identity as being athletic and physically able. He acknowledged this identity as a loss, contrasting the past with now for instance "*I was doing the dead lift, 150kg, you know, one or two, and then you'd like pass out…I think my personal record is 350kg on a leg press…And to see my legs now, I've lost so much muscle mass.*". This was followed up by identifying what cannot be done currently, one aspect was having no strength to move his leg inward. These experiences affect him as he states *'that's hurting me, nor hurting physically, that's hurting emotionally at the moment....literally got nothing there, but I don't know how to do anything about that*". P12 talks about the possibility of going back to work a job which is enjoyed, but there is an element of being unsure if that will happen and identification that it depends on progress with his arm and walking which is unknown.

An important and changing context for the return to normal was given by using reference to time. The first reference to time was the open future. For instance, P4 states "*I just know it's going to take a while, but, like, I'll be back good as new at some point*". Another example is from P18, who states "So I'm thinking my leg will be the same [restored or fixed], once I get through it. Like I said, little wins make a big, big difference at the end of the day." A second reference to time was an adjusted future or extension of expectations. For instance P1 states "*a couple of weeks and I'll be back to normal. And now it's gone from a couple of weeks to I'm… if I can walk before Christmas I'll be laughing*." (P1). In a similar way P20 states "*I'm just sort of slowly recovering at home now but it's going to be four to six months before I fully recover I think, or could be longer*." A third reference to time and the future was linked to outcomes of surgery. For instance, P17 states the "*hip eight weeks, the ribs might be slightly quicker*". The fourth and final reference to time was given this included the potential for a problem in the future, for instance, P17 recognised that there could be 'something' wrong identified during recovery which, will, in the future prevent him doing his job, he identified this within a context of having a lot of injuries that needed to heal and that this took time and would occur in an order. Alternatively, P15 identified "*they [HCPs] made me aware that a year down the line things can still go wrong with the grafting*".

Master plot 3 time point 1: Wanting or needing to look forward and accept; the birth of the quest

The basic plot line for the quest narrative was that I can embrace the present circumstances and use them, adapt and a valued future is possible. Slight variations in the quest narrative appeared to hinge on how acceptance varied between individuals.

(3) a reframing presentation of what had happened, essentially it is not how others see it, it is not being disabled because they are able. P14 who states "*other people might think of me as disabled. I think I'm extremely able. Yeah. It's a matter of attitude, isn't it?* " He goes on to illustrate an accepting nature of his attitude that makes everything useful or useable, akin to an adventure narrative. He considers how he embraces difficulties or challenges is stated "*Healing, physical healing takes longer when I'm just there depressed.... And, of course, having a good time is much more enjoyable. Yeah. There's always a silver lining. And if you can't find one, you can always create one....It's always been the same. I always was of the opinion, well, if life gives you lemons, make lemonade*".

(4) the importance of working with what is possible at that moment in time. For instance P1 states "*I always just try and think about the positive of it. If I've hurt my knee, then I'll think okay, now I can do some more stuff with my upper body and just keep that in mind, in a physical sense, that's the way I sort of look at* it". (P1).

(5) an attitude of problem solving. For instance, P12 identifies the importance of getting around problems he states "*I'm doing okay. I'm quite determined to get round it. And I can get about on my crutches quite good, in fact, I feel sometimes that I don't need these crutches*".

P20 and P18 identify the importance of changing as a result of what has happened illustrating the quest auto-mythology sub-type plot. P20 states *"" I just feel lucky to be alive you know and its completely changed my life, my attitude, you know I just want to look after myself more. I've lost, my weight is down, I've never been so… my weight has never been so low.* ". P18 identifies a transformation and quest auto mythology as part of his narrative because of the change that has occurred he states "*he has to 'learn to live with the injuries*', *'good clean living, eating healthily....get as mush rest and stick to their [health care professionals] advice*"

Factors which influenced acceptance
The ability to accept or embraced what had happened appeared varied according to specific strategies that enabled acceptance. This included (1) past experiences and knowing what to expect and there for accept, for instance, P14 and P17 identify past experiences and injuries which influence acceptance. For instance, P14 identifies accidences from epilepsy he states "*Not again. (Laughter) Yeah. Here we go again….I was not frightened at all. I know what to expect.*" (2) identifying acceptance as not something there is a choice about. For instance, P10 identifies having acceptance to what he was being told without choice.
(6) there was an attitude of holding expectations lightly, for instance, P24 identifies marking off what may not be possible in terms of function and the future. She states "*I am aware that that is maybe not possible, but I'm trying to sort of keep quite a neutral mindset, not worry one way or the other because I don't think that that's helpful*"

Time point 2 analysis 6 months

The restitution as broken down by time and action

The restitution was referred to in two ways, by those who had established a great deal of what they referred to as normal and those who could see restoration in the future and expressed that as a desire.

The establishment of a majority of normal activities

The restitution was most easily expressed when most of normal activities hoped for were established since last conversation with the researcher and only a few limited changes were identified as needed or wanted. Some participants emphasised the experience of achieving normal activities, for instance P4 states "pretty much back to normal. You know, I wouldn't say don't I notice it, but I notice it 1% of the time". P10 states a return to "the little things of normality" including driving a car, being able to make the stairs. The idea of normal refer to activities of daily life with some functional limits still required. Examples of what was required included more strenuous or difficult activities or functions, for P18 this was not running yet. The remaining changes were identified as either not problematic (could live without them) or as possible to overcome or change in the future.

The restitution and wanted futures

The idea of being restored as normal could be expressed in different ways but often included a reference to activities the participants used to undertake. For instance, P1 desired to get back to the gym, swimming and planning with his children. P24 identifies specific goals agreed with his physiotherapy including yoga, swimming and being able to run 5km. P12 identifies the want to get rid of specific activities like waking up in the night to 'mess around with a catheter' or 'not having to visit the hospital to see the physiotherapist' the reason for this is because of the impact on the family that these activities have.

Being get back to normal could be expressed as a percentage of what they could do before the accident. For instance, P17 states "*I could probably get back to 90%*" and two (P20, P24) participants mention 80% to 90% recovered including. P24 states "*[I am at present] about 80% function may be now and I'm hoping to get back to 90%"*. P18 talks about getting back to '*how he was*' although identifies that this is dependent on setbacks. The idea of a percentage of normal regained was often provided with a timescale. For instance, P7 identifies a time of 6 months for walking, P20 identifies 6 months and he 'should be in a good place', P10 identifies a time scale of 12 months to hit targets. P1 and P4 identify a time of 1-2 years and 2 years away from getting back aspects of normal living. P17 doesn't provide a scale but identifies no problems with looking forward as he sees the injury as being fixed. The idea of being fixed is abandoned by P14 who focuses on getting to a better place than presently exists with activities, a major goal is walking distance as he used to.

Unwanted futures, moments of sadness and tragedy

Some participants identified seeing or experiencing outcome which contrasted to wanted futures and restoration. P1 identifies how the CT scan reveal that from surgery nothing further could be done. P1 identifies the point at which further change was not possible but the 'rollercoaster' that led to this, stating health care profession would at points say *'we're going to fix this, no we're not, yes we are, we can do this, no we're not*". In a similar way P24 identifies he previous ability to hit every goal anyone set for him, but more recently that 'there are no more goals to hit', demonstrating less of hope and change in the future

For three participants current experiences and problems means life was more difficult and not what was hoped. P12 identifies having a catheter, he states "I went there two or three times, finished up having a catheter because I couldn't pee, and that is still ongoing, which is a bit of a… it's sort of slowed me down a lot really because A, I find it difficult to do the exercises with it, because the two don't go together very well." This impact was meant to be resolved with the catheter coming out but recently that was identified as not possible, these experiences illustrate a sad or regressive or tragic narrative as he states "So that's kind of set me back. Just when I thought I was going so good and I was so chuffed with it all, it's all sort of gone to pot." slowness of change and the outcome being dependant on the next appointment he states "it's still very painful to walk and things seem to be deteriorating again. I will find out next week when I have my six weeks appointment with the trauma clinic. I was told before if it's not better, the only option is to have one or both knees replaced ." He follows on with a lot of detail around what is not possible or what cannot be undertaken for instance he states "I'm living life in the slow lane now".P15 highlights the impact of his current situation as he looks forward. a fear that he may not be able to walk normally again, but he recognises the needs he has currently and limited mobility where he needs an air cast boot. He states "*sometime of the day I mean I feel positive and try to keep some positive thoughts about it. At the same time, it's like, well, I do get thoughts of like I'm not going to be to walk again. Is my foot ever going to get better or will it need an amputation in the years ahead, what my life will kind of be in a year's time and what adjustments will I need to make if I do need to have an amputation done? And yeah, it's been quite overwhelming with all that*.".

Getting to normal and the need for the action narrative

Taking action to get back to normal or improve and access to a more certain future was expressed by several participants. The idea of action was closely linked with the need to be independent (P14). The action narrative included an expression of taking ownership of the situation (P10) or was provided by stories of accomplishment, for instance to access to work (P17), P17 states "*like I said, driving is no problem. I went to do a job, an interview job in Holland with our photographer last week and I drove all six hours back, no problem*". The action taken for individual could result in negative outcomes for instance P17 identifies feeling achy because of choosing not to wear a brace recommended by health care professionals for his back. P24 identifies the experience of pain and P7 identifies being put out of action due to a fall. For P4, undertaking difficulties activities result in some negative outcomes and that is accepted. He states "*this weekend I did my first sort of intense riding. But I was so sore; and I'm still sore, actually, three days later. Just the muscles are, you know, just really, they worked really, really hard. So, I think it's really important that I stick to… continuing… and also walking, hill walking and stuff like that*." P10 identifies the importance of lying down with pillows to help the pain stop.

Activities can be done where problems or barriers are worked around (P8) and this can be overcoming challenges for instance P14 states “*the ambulance people brought me back to my home, they were absolutely gobsmacked up was up the stairs in less than a minute. I have very strong arms to bum shuffle*”.

A lot of participants were able to mark of limits to activities or activities that they would not risk currently which represented exceptions to the action narrative. Limits are selected or activities like standing on a stool, drop height from steps or putting on a sock (P10), standing on ball of his feet (P12), range of movement due to pain (P14), or avoiding movements like bending or twisting, trampolining, heavy lifting which will be avoided until a scan gives the ‘all clear’ (P17), or avoiding specific activities on the farm (P24) or whilst at work not being able to kneel down as they could not get up again. Avoiding activities was identified with a specific reason by P8 to protect themselves e.g., *“I don’t want grief and aggravation and stress. I know I’m lucky to be here, and I want to feel like I can make the most of what’s left*."

The quest narrative

Acceptance as findings solutions. P7 identifies the need of finding ‘a path round’ a problem and that activities (and normality) continued "as long as there are within human ways of doing it".

P7 also identifies the need to observe the environment more now for instance awareness of pavements “banky pavements and stuff like that are quite a big thing for me….Especially in the wheelchair”. P8 identifies the importance of resting. P18 identifies that the a more recent accident paused ‘everything’ and could accept this.

Acceptance that that past as it was may does not return completely. P8 identifies that you cant ‘recover from everything completely” and identifies the importance of acceptance towards his pain that may not change. P8 provides a clear understanding of the change occurred and that further change is unlikely, for instance, he states “"It’s fairly rubbish as to what it can do, but it’s a better option than having no arm, which obviously, was a distinct possibility at the time. So yeah, I’m quite accepting that what I’ve got is adequate; I can work around it. I’ve got limitations with it, yes, but you know, at the end of the day, there’s people a lot worse off than I am”

P7 identify the idea that you cannot replace things how they were. P12 similarly identities that there is nothing he can do to change the situation that he just ‘moves on’. P15 identifies that find 'alternative' ways to be more dependent on himself including how to get shopping when going outside is not possible and that "*now I have to plan everything and plan my food, my weekly intake of food….its been hard adjusting to all that sort of thing*". P20

identifies that he will have to live with the changes, despite health care professional encouraging him to maximise movement in his, his acceptance is about the future and that change has become uncertainty. He follows professional advise but doesn't know if change will come.

Acceptance as making the most of what is possible. P8 identifies those that are worse off than him and that he needs to "stop moaning" about his injury and appreciate what he has and make the most of what he has now. P24 has as similar philosophy but rather sates "I set myself the goal of being the best I could be". P14 illustrates he need to accept with the benefits that arrived as a result of the accident, he states ", *'it is no good crying over spilled milk', and 'if i cant change things, just embrace it and get on, because I have a fair few years behind me...my experience is that when one door closes, another one or two open...So i just embraced that...I actual now live in life in the slow lane. But I made friends that I never thought I would make, so there's always something opening. So one of the next-door neighbours, we just said, 'hello' when we saw (each other recently), we never talked (before), And now he's my best friend now'*.

Factors that influences narratives

Individuals were generally more positive about the future when they could recognise the benefits and value of engaging with rehabilitation and exercise (P12;P20;P24).

Moments that require reconsideration of experiences

Pain whilst performing and activity, limited range of movement, and less strength appeared to be associated with a perception and risk and concern around undertaking specific activities. Concern was around the potential to cause further damage and pain and meant that some activities were avoided. For instance, P1 avoided walking and 'doing too much' or 'catching myself out' as well as not doing sports where there is pain and known when to 'take a step back' or limit activities because of pain. P15 and P18 identified the possibility of damaging their joint through performing activities. P15 identifies the risk and perceived problems associated with a slip and fall. P18 identifies the risk associated with sport and concern around injury. There is a hope that there will be strength in the muscle to prevent this. The risk, worry and concern act against the individual's ability or want to engage in specific functional activities such as taking the trash out. P8 identifies the impact of fatigue and pain which adds to concerns around movement. P8 identifies finding, what he considered relatively simple tasks, as now being challenging. P4 states that he ended up pushing the body too far in rehabilitation and has had to learn to "*respect the recovery process more*".

Moments helped action and the system of rehabilitation e.g., having a prosthetic fitted (P7), physiotherapy to work on body part and counselling (P8), or feeling that they can do their own plan, routine or rehab when limits from the injury do not prevent this (P15). P17 identified a moment of progression stating “"woke up one morning and the ribs were fine." and continues "so I thought well, I’m not in pain now." P17 identifies once medication was reduced and pain was absence it was a sign that ‘everything else’ was healed.

Moments that were aided by internal considerations. For instance, P10 talks about the “tenacity and determination to keep going” despite the situation getting to him. P17 states “the more sport you do, the less it fatigues”. Planning was identified as important by some and small wins are a focus (P10). Goals could be being fitter than they were before (P17). Problem solving was important to allow activities and rehabilitation to continue (P20). However, aspects of life could change this because of life challenges like being tired after shift work or having children (P24). Moments of understanding the journey to come P4 states they told him that “recovery istn a straight line…sort of in one direction” that there will be bad days and weeks and that they were “good about helping me manage my expectations…deal with my frustrations” and he also mentions positive results from a scan and the big difference that was.

The stories of others

Reference to stories of others for P1, he identifies people who had their accident 8 and 20 years ago who are now, pretty much back to ‘normal’. P1 also mentions “people that have been told they are never going to walk again. Next thing they’re winning an Olympic gold medal”. P10 identifies someone who had an accident 30 years ago as an idea of how long it took for improvements. P7 identifies watching “above knee people…and I think…if they can do that…ride a bike, ride a motorbike, climb mountains [I can]”. Other comparisons are made from attending events after hospital of people who have a lot more limited movement that she does for instance ‘using a figure to move a wheelchair. There is a want not to be like others who “are living with their injuries, but let them hold them back, I Don’t want to be like that” (P1). P20 identifies a work colleage and times when his injuries plays up such as too much walking and in cold weather, but also identifies time needed for the swelling to go down, which he links to his own conditions saying six months and I should be in a good place.

Future uncertainty

Uncertainty was present in different ways for participants. For P8 it was not being sure about how the injury was repairing itself and if that was on course. For P10 the seriousness of his scan and the fact there was no commitment or conversation around potential for recovery he was left not knowing what the future would be in terms of activities He states "I don’t know if I will be able to run or not, I’d like to, even if it's a jog. I don’t know what to expect…I expect I might have a limp”. P15 identifies that health care professionals do not know what is going to be needed in a years time

and were not able to identify what is needed. P20 identifies his age as a contributing factors and states. “Am I going to fully recover or is it something I’m going to have to live with and stuff like that?". P24 identifies the uncertainty of not knowing what the future will be like and the emotions that are associated with this “*I have had real ups and downs of feeling like is this where I am at now, and it’s not going to go any further ?”*. P10 also identifies emotions in particular the feeling of fear. P10 identifies a fear of the future being like the present, despite as of yet, not one saying this, he states, “Well I guess if someone actually turns around to me and says, this is as good as this is going to get, which I am very fearful of happening. But no one has said that to date”. One way to deal with uncertainty was expressed by P14, he focused on a more general openness to experience and possible futures is identified by P14 who states "I know there’s always a door opening again that I didn’t know. It makes me kind of nosey, curious, what good is coming in my way? Because something good is always coming my way then. "

Moments of social support

Moments where support was provided are identified specifically as positive influences on the individuals story.

Moments that created a positive change and freedom P1 states “It was almost when they [physiotherapists] said, “Go live your life,” it was just a switch for me.” This was identified by P7 that that a physiotherapist who was private had protocol to get the “person back to full strength as quick as possible” but also identifies needs like a new wheelchair and a stair lift. P18 identified how the osteopath was positive each time he states “Every time I went there, everything seemed to be bad, but when I went to my osteo, everything seemed to be good“. P20 identifies timing as considered by others who had a similar injury and from experiences in physiotherapy of encouragement to perform exercises.

P7 identifies the positive changes from her private physiotherapist.

P7 identified conversations with an NHS occupational therapist [OT] stating “I got a phone call from her [OT] saying, “Sorry, you don’t qualify for a stair lift……” I said, “[name of therapist], you are now putting me at risk of falls”. Other negative instances include form P14 of only having one 5 minute session of physiotherapy after which he was sent to care home for people with dementia which he signed himself out of and de-registered from his doctors. P15 identifies feeling left alone once discharge happened with no one providing reassurance he found it negatively impacted his wellbeing. P17 identifies problems with a specific surgeon who, he reports as being identified as creating problems with others who went through a similar experience. P17 also highlights problems at the point of discharge in not being able to adapt to his home situation and requiring support during this time for instance he states “I live in a sort of 60s semi and there’s no downstairs loo. So they wanted me to be chaperoned up and down to the toilet.". P24 identifies missing elements from the NHS like never getting an NHS bed, or seeing a district nurse and having to do things themselves such as going to get a dressing renewed. This is contrasted to a private physiotherapists which was identified as a turning point.

Support from family was identified as essential for instance P10 states “if I was living on my own it would be a different story. I think having the support around me has been essential, especially coming out of the, you know, sort of being an outpatient. Initially I was very scared about coming

home”. P14 talks about people in generally who ‘actually want to help me when I have heavy things to carry…so nice”. P8 identify the value of support but had told his story enough and he identifies the most common questions that people ask when they talk to him about the accident. He states “it becomes a 20-minute conversation about something that I’ve repeated quite a lot of times now. And yeah, I need to look forward, not back”

P14 identifies poor care experiences including limited physiotherapy experiences 'one session that lasted 5 minutes intially' after which he identifies being sent to a care home for people with dementia, which he signed himself out of at the same time he was de-registered from his NHS doctor. P17 highlights poor service with a surgeon and gives past examples of poor results for patients. P10 identifies that on reflection he came out of hospital too early, this isn’t attributed as a fault of the NHS, but he does admit to being scared about coming home and that without the support of his spouse then it would be a different story.

P24 identifies being fearful of driving as something that has impacted on experiences of driving she states "*I’ve had a few incidents of crying in the car and things, especially if the kids have been fighting and I haven’t felt just safe*",

Final time point 12 months

Restoration narrative and returning to normal

The major plot of the narrative was that living normally without significant achievement was established. The idea of restoration was primary focused on returning to normal activities, which mean activities that could not be achieved were often considered as non-essential or that could be lived without. This could be relative to any one person for instance P20 states "*Yeah, I mean, I can't run or anything, but I mean, I'm not into sport or anything like that anyway*.".

P4 states that he should be able to climb the stairs following a workout but currently cannot or would like to be able to jump from a high without hurting his knee but cannot currently. P7 identifies skateboarding as one activity which he will struggle to do. Some participants were not quite as this stage, but still referred normality. P10 for instance talks about "some normality creeping in" but not quite there with factors like bending down. P15 talked about getting a '*bit more normality back'* as a direct result of recent progress, however, that he still relies on others for help. P17 identified needed improvement in flexibility and muscular build up.

Participants talk about living a normal life, participants were able to identify what this means in terms of the future and in terms of specific outcomes that are assoicated with normal activities. For P4 this meant living a 'normal life' now and that the injury didn't affect 'daily life now', but that to be completely restored will take much longer than first thought, he states "*I was going to be okay by Christmas. It's now been a year. I'm obviously still not okay. So, that's probably the biggest wakeup. So, I've times that by about 10 [years*]". For P20 normal was described as being "*almost fully physical and mentally recovered from the injuries*", he associates this with being able to walk, but also that certain limitations that exist like having numbness or swelling as not affecting this. P7 identifies doing activities in his workshop which he never thought possible. P17 identifies being able to hoover his car and cut his toenails.

People provided clear identification of limits of movement, and this was associated with normal activities and function. For instance, P1 states "*Putting your pants on. Putting them on, getting out of the shower, standing up from a chair, walking up a set of stairs. Not worrying how you're going to roll out of bed and if it's going to hurt when you get out of it. Going to, walking to the bus stop. Just general sort of stuff that's not going to be in a way that you need to be worried about the terrain you're walking on. That's normal for me*." P18 identifies not being able to go up a ladder or do netball and take such heavy landings currently. P24 identifies not being able to 'run after the kids' and not being quite up to normal ability. The importance of more strenuous or challenging tasks varied for some it was important be able to do sport, mountain biking or horse riding or motorcross (activities at times that represent how the accident happened) where for others that was not important. P7 identifies how age could influence this stating "*I'm an old sod, so I don't have to worry so much about public image and stuff like that, pulling women, going on holiday with the lads and all that sort of...when you're young, you think... you're going to be sub-human if you've lost a limb or something*". P12 identifies the impact of an additional challenge of having a catheter which made getting back to normal less possible. P14 identified problems identified within a scan which could explain

why getting back to normal may be prevented, he states "*could see that the other leg was just as bad as that one. So, it looks like a minefield of bones" and when considering a scan with health care professionals he notes "a piece of bone sticking out" after which it is agreed that it can be removed surgically.*

Quest narrative

The basic narrative plots appeared to be represented in three forms;

(a) the first plot was that the participant had identified a different path moving forward or established a subsequent phase they had entered or that had opened up for them. For instance, P10 acknowledges that no further improvement was possible from rehabilitation, but he feels better about it. P1 talks about a different path taken stating "*different path [is] taken…recovery means like in my mind I was going this way, my accident made me go this way [a different way], but I still want to keep on going forward but just on a different way*"

(b) the second plot was that there is still enjoyment of life and activities to be had, or an ability for positives to be seen. For instance, P14 states "*I'm a glass half full person…. I met some really nice people I wouldn't have met otherwise*". P12 consider how he views different problems stating "[I've got] *numbness. In my back…that's getting better…my arm…I've lost all muscle in it…but it will be alright… I'm a bit restricted with that but at least I can get about*".

(c) nothing can be done about the problem. For instance P24 states compared to when the injury happened "*I'm almost more negative but not in a very bad way but like now, I'll be like, "Mm, yeah, I can see maybe that's not going to be great but it's how it is*.",

The quest narratives were supported often by an individual's ability to adapt to challenges e.g, making changes to the shoes you wear and finding ways to do activity your own way (P1) or wearing crutches (P15), and finding the best you can be and maximising the present (P14) or being proud of being disabled (P7). Interestingly for P7, a few moments when a health care professional provided statements the related to recovery including, a statement around the fact the prosthetic leg could not be seen as a normal leg or treated as such, he states "She said to me jokingly, she goes, "*Look, you've got to realise that that's not your own leg, it's a prosthetic leg…. I needed someone to tell me that because sort of like, the way things were going, it was like, everybody was saying, yeah, it will be good as new*." and a moment when he was told there is 'nothing more we can teach you', in effect he needs to move forward, was again taken positively and aided the quest narrative.

The quest manifesto could have been created by three participants although all three stop at identifying problems with the health system and do not identify an action plan on how to change it. P7 identifies problems with the insurance system and systems in the government that didn't work and having to interact multiple times to get continued support. P14 identifies signing himself out of a care home, he also identifies not being given the correct medication in the care home and not being believed about his capabilities by the ambulance staff, he stated "*They [paramedics] said, "No, you can't," "Oh yes, I can." "Well, you show us." And I was up there no flat…It was just under a minute to get up that flight of stairs with a bum shuffle*". P17 identifies that scans and physiotherapy took a long time to arrange, but identified the impact of funding on these problems rather than identifying staff.

Action Narrative

The basic plot of the action narrative is by undertaking action (including rehabilitation, exercises, accessing treatments) improvements are possible. The narrative is supported and determined by doing activities which provide a learning environment. This can be quite strategic for some participants, for instance P7 undertakes two walks a day and at weekends ensures walking in '*all sorts of terrain and all sorts of weather*". P1 talks about taking initiative whilst waiting on PT stating that he is "working on posture and walking and things like that". P10 identifies a gradual process that results from action, he identifies being crutches free and now is focused on walking more. In some instances, the value of action and experiences of successful movement can be attributed and be supported within a specific event. For instance, P18 "*even my fitness improved from my first game back to my second one, it was amazing, so I'm not as worried as I was before*".

The action narrative is considered by looking forward, being future focused and having an ability to take steps forward and the individual may limit time given to looking back. For instance, P18 states "*when you're obviously recovering, everything you're doing is to look forward. So you're not really thinking about how you felt before".* Action can be aided or determined by a specific goal, identification and need to get stronger than before (P4; P18), walk longer and further (P14) or that by doing the level of activity will make it easier in the future (P7). The action narrative is supported by persistence and through acknowledging difficulties and challenges that reinforce an ability to change cycle. For instance, P14 states "*I'll practice that more and more and more. I know it takes time, but it does seem to work*". P24 states improvements from exercises like Pilates and swimming which have allowed her to run, which she states, "*is very painful but I can do it and it will get less painful*." The action narrative requires the individual to take responsibility for instance, P17 states the need to address his remaining limitations are up to him. When only a few or small improvements are left this can aid the narrative. For instance, P12 states "*just need to keep on keeping fit and keeping, you know, doing the best you can… just got small things to get adjusted and then I should be fully recovered*".

Individuals could identify changes made that allow action to happen, for instance P7 states *"I've got the car now, so I'm going to gym twice a week*. In a similar way moment of improvement help create motivation for the future P15 states "*knowing that there's a slight bit of progression, knowing that*

there's a possibility that it could get a lot better, I think that's been quite positive." Another moment mentioned which allowed a significant change towards action was the prothesis fitting. The narrative is supported by different forms of action taken, for instance, P15 talks about taking medication for cartilage growth, but also talks about private health care, physiotherapy and surgery. P7 identifies quicker results due to private health care access. For P10 a warning was identified from stories of others who did not undertake action. This state "*speaking to people who've known other people go through similar experiences, albeit probably a bit older than me, they're saying that someone's had a hip replacement and didn't do the physio or refused to do the physio and never got back to full mobility ever again. So that is a bit of a warning sign as well, really*".

There were specific stories and experiences which acted to prevent action or could be considered as an anti-action narrative. P14 has been able to overcome pain by cycling but could not cycle to all locations needed for instance he couldn't cycle in a train station or on the platform. P10 states that requirements from having a young family come before the ability to adhere to rehabilitation. Actions appeared to be helped by access to private care and could be hindered by state services (P24). P8 identified that 'surgeons and solicitors' prevented going back to work as part of the insurance process, so prevent action in that sense. Pain was identified by P17 as stopping action and P20 identified that they had no goals as part of the interview starts thinking again about them, stating "*stopped thinking about goals - think it wouldn't be a bad idea for me to continue some of those just to continue to tone the muscles in the leg in particular"*

Pain as an influence on the individuals ability to access normality and action

Time point 3

Pain was identified as significant by some individuals. This included pain during activities for instance P14 stated he could not "walk one step without pain" and P8 identified pain associated with tasks like picking up a computer, locking a door (P8). It could be significant that it meant conversations could be difficult as the individual was constantly "thinking about the pain" (P4). Limits of activities or movement function is identified "*"so I'm pretty sorted in knowing what is or isn't possible and what is possible providing I accept it's painful to do it. " (P8). P24 identifies the difficulties psychologically of dealing with the experiences of pain when looking forward, she states "can be a bit psychologically difficult to deal with because you're sort of asking yourself, is this something I'm going to have to keep forever?"*

The experience of pain would indicate limits of activity and in examples this was transferred into a risk assessment and implication. P17 states "if you stretch of bend or do something particularly tricky with the body, you get a little twinge that says, "You're pushing a bit hard on that bit." P24 identifies

being 'hesitant' doing some forms of yoga or positions, climbing fences, driving for more than a few hours or doing an activity which leads to discomfort afterwards. *P8 identified the risk of a slip or a fall, that pain is a warning that damage could be done and as a result of this he states "I have be a lot more cautious than I would have to be normally". P8 states that there is no warning or control over how painful a moment may be which means he only does an activity if he "wants to do it bad enough". P14 identified riding his bike as dicey and as a dislike but didn't didn't worry about the risks because of past experiences, stating "". I might have another fall, you never know, it wouldn't be the first one with the epilepsy." P15* identifies risk and worry about having a fall as he looks forward.

Sad or negative moments

Rather than a strong regressive narrative that had a clear plot around difficulties, participants identified a reflection back on moments or a more detached commentary of the importance of not being negative. Dwelling on what has happened previously and identifying a meaning of being negative. For instance, P1 states "*I feel as though if I sat down and dwelled in all it, it's just going to make it worse*". P18 shares a similar view, stating, "*as soon as you get frustrated and negative about it all, it all becomes a lot harder. Interviewer: …Do you think that mentality almost was quite important for you, looking back over your recovery? Respondent: Oh yeah, 100%, I think it's important for everyone really, sport or not, if you're not positive to getting it back to how it was, it's just not going to [happen]….*" Moments of difficulty was identified with reduced improvement or experiencing a plateau in change for instance, P4 stated: "*I really started to do really well with my initial recovery, then I just kind of like plateaued, almost went backwards. That was really hard*." P24 states a period of the summer holidays where she felt low. She states "*it was really tough, it felt like I'd had a sort of backwards step, which was really unsettling after having had kind of generally kind of upward progress before that. I felt pretty low then*." P12 identified an additional problem of having a catheter and the need for that to be removed and that the impact of the catheter before it was removed was identified as something which "gets you down" and that "*if you'd have asked me that question [how are you getting on currently] two weeks ago, I'd have said, It's not going very well*". The reason for this was due to frequent hospital visits caused by the catheter and need for antibiotics. Other moments included the impact of a scan assessment for instance P17 states "*"It wasn't great news at that point, it wasn't fully fixed and all the rest of it."*. Other factors which were specifically mentioned were reminders of the injury from others for instance P12 states "*everywhere I go, everybody, you know, they're only being kind but they constantly go over it, you know. I just want to forget it, really, you know*?" .

A more permanent sad narrative was identified by P8 identified a lack of progress and a limited outlook, stating "it feels…nil progress now", he identifies feeling "upset" and affected by the anniversary of the accident recently. He identifies that a grieving processes "*it's sort of comeback worse I suppose. I thought I was over the hump and more accepting and that's difficult now because I think the realisation, the reality is that we're pretty much as good as it will get. And in some ways, it's like worse. My back is really painful and the pain*". P8 also talks about the impact of limited progression over time stating ""*I knew I'd never get back to exactly where I was, so recovery was accepting where I am. But now as time has gone on,*

with even less improvement over the last three/four months, that feels more difficult to process because the progression of repair and recovery is not there now. So now I'm at that stage of trying to just accept that it will be what it will be."

Additional summaries were made

Initial early reactions and the birth of stories

The recollection of the moments when experiencing the accidents and injuries and the moments that followed provided the starting point for identifying meaning in relationship to time

Time point 1: Reactions at the point of the accident and disembodied descriptions

The disembodied narrative was consistently used to describe the events of the accident. The basic plot is that of a tragic accident which is identified in details and reactions at the time as they look back. Participants often started the interviews by describing the accident. The description of the events was represented by a disembodied narrative describing what happened in detail and clearly articulated. At the point of the accident there was a sense of shock. For instance, P12 stated "I remember feeling strongly like, "this can't be happening". P15 could not see his motorcycle boot on his foot and that his foot was dangling off his leg. P4 was initially taken to a hospital away from his home. He locates this experience by stating "I didn't know anyone, there were no visitors allowed, I have zero connection to [name of city]". P24 identifies being in a state of confusion about what was happening, being told afterwards by others that she was slipping in and out of consciousness.

Several participants identify the significant and overwhelming experiences of pain. The experiences of pain are expressed around surgery, involving having to put up with spasming muscles and 'horrible pain' and waiting on medication in hospital to provide relief for the pain and the urgent need for it before it arrived. P18 identifies detailed pain experiences during the transfer to hospital, stating "I had to get my mum to tell me every time there was a bend…Every time I tensed my leg, it was so painful. It was just another type of pain; it was horrible." P20 identifies the seriousness of the accident with broken bones in a short way, the pain was identified but again described objectively and briefly. For instance, he states "Initially they give you a lot of morphine to help with the pain and then gradually after about five or six days they took the lines off me and just gave me morphine orally, if required." and later states initially it was pain like he had "never experienced before".

Master plot 1, time point 1: Negotiating the present and a need for action narrative

An action or need for action narrative appeared to be prevalent across participants. The basic plot was that if I can engage in rehabilitation right now, or I act in the present then a more independent future or, future with better outcomes is possible. Key components of the narrative appeared as having a focus on making small gains or taking small steps to improve and this is to guard against being demotivated which was recognised as a real possibility. This narrative was created in a context when past progress has not been undertaken, and the certainty of future outcomes and progress is limited. For instance, P4 states "*I need to do exercises and do that and really stay motivated. So, I think just staying positive and, like, focusing on, like, the short-term day-to-day, the exercises, I think that's helpful. Because…people easily get demotivated*". A core objective of the narrative was being able to take action that could allow access to potential change efficiently. For some this was contrasted against an idea of dwelling on the problem or the past which could prevent it. P1 and P12 identified the importance of an intention to change, or a need or commitment to change. For instance, P12 states "*I've just got to get better, you know, determined that I'm going to push through it."* and later he says "*to be independent, I've got to put the work in*". P1 states "*if you don't see everything as a challenge and if you don't try to accomplish it, it's not going to be worth it. And if you're sitting there going, my recovery, dwelling on it or anything else like that, then you're not going to get through it as quick as what you want to*". Other components of this narrative included an idea of a positive mindset and keeping motivated, P15 states "*if you have that positive mindset, you're going to physically do whatever you can do to make yourself better*" (P15). Another aspect was looking forward to the next step or change. P8 identifies the value of surgery for moving things forward He states ""*that morning and the day of surgery, I was looking forward to the next one to get things done*". This narrative may also be supplemented by a need and strong motivation to accomplish a specific task or goal. For instance, two participants P7 and P24 had a very specific intension of getting out of hospital as soon as possible. P24 arranged home adjustments whilst in hospital and, at the same time, engaged with health care professionals in a directive way. She stated "*every time I saw an OT, I flagged them down until I found someone that was willing to work with me*". P7 identified a goal of "*not to get comfy*" in the hospital, this contrasted a view of others in the hospital who were perceived as comfortable to "*do their bedpans and watch all their TV*".

The narrative was supported by moments of overcoming challenges. For instance, P24 illustrates action in overcoming challenges stating "*I have been back in to see the consultant this week, on Tuesday, but again, I had to do that myself and get myself in the car. We had to drag me into the car and drive me to [hospital location]*". This narrative was helped by specific moments of change or accomplishment early on during rehabilitation. P10 and P17 identified the need for achieving small changes which provided '*something positive'*. P1, P17 and P18 identify specific moments of change, this included taking first steps standing up right for P17, and for P1 it was walking, he states "*I didn't think I was going to be able to and then all of a sudden, within two minutes I was walking*" (P1). P18 talks about identifying a turning point, stating "*[I] did my stairs, and then got a wheelchair back. So that was...Thursday was my turning point; my good day*". P14 identified the importance of steps and states "*once I've achieved one [goal], then I can do the next one...Do the same as before, a little bit faster*".

Attempting movement and rehabilitation could also identify limits of action and possibilities in the future. This experience demonstrated that the process of change was not linear and this could act to limit and challenge this narrative master plot. For instance, P1 identified not being able to navigate stairs and the act of trying made him realise it is not possible. P18 identified low energy levels and limited allowance of movement for instance he states "*I wasn't allowed to do any more weight bearing than just toe touch weight bearing*". Other aspects which challenged the idea of action occurred if participants were awaiting an assessment result or for change that could happen in the future. This could mean that life and the future was on hold. For instance, P4 acknowledged the physical limitations current and what they mean in within his present situation. He states "Well, it really is just the physical limitation, right? Like, I cannot

walk down the block; like, I cannot imagine getting on a plane and walking through an airport. Do you know what I mean? Like, carrying a whatever. I just cannot; nowhere close to that at the moment”. Other examples provided by P1 and P12 identify difficulty with day-to-day tasks such as taking a shower, going to the toilet, getting dressed and other limitations in movement or activities. Finally, following the accident P17 identified that he would be more risk adverse which could narrow what activities would be engaged with and impact the future.

Master plot 2, time point 1: The future and establishing what is normal; the birth of the restitution

The focus of restitution and basic plot was that I will return to some form of ‘normal’. The reference of normal was focused on activities and relationships, roles or social identities previously assumed. The way the narrative changed was around what was represented as ‘normal’ for each participant. Normal was represented in the following ways: (a) normal as being ‘like new’ (P4) as in a being fixed, or, (b) normal could refer to activities, specific movements, function or specific roles. For instance, P10 identifies a need for restoration towards being mobile for his children, he states “*as long as I can get out with my kids, you know…just stuff we did before , that's all really*." The importance and realisation that normal could not be a straight fix is illustrated by P10. P10 acknowledged his past identity as being athletic and physically able. He acknowledged this identity as a loss, contrasting the past with now for instance "*I was doing the dead lift, 150kg, you know, one or two, and then you'd like pass out…I think my personal record is 350kg on a leg press…And to see my legs now, I've lost so much muscle mass.*". This was followed up by identifying what cannot be done currently, one aspect was having no strength to move his leg inward. These experiences affect him as he states *'that's hurting me, nor hurting physically, that's hurting emotionally at the moment....literally got nothing there, but I don’t know how to do anything about that*". P12 talks about the possibility of going back to work a job which is enjoyed, but there is an element of being unsure if that will happen and identification that it depends on progress with his arm and walking which is unknown.

An important and changing context for the return to normal was given by using reference to time. The first reference to time was the open future. For instance, P4 states “*I just know it’s going to take a while, but, like, I’ll be back good as new at some point*”. Another example is from P18, who states "So I’m thinking my leg will be the same [restored or fixed], once I get through it. Like I said, little wins make a big, big difference at the end of the day." A second reference to time was an adjusted future or extension of expectations. For instance P1 states “*a couple of weeks and I’ll be back to normal. And now it’s gone from a couple of weeks to I’m… if I can walk before Christmas I’ll be laughing*." (P1). In a similar way P20 states "*I'm just sort of slowly recovering at home now but it’s going to be four to six months before I fully recover I think, or could be longer*." A third reference to time and the future was linked to outcomes of surgery. For instance, P17 states the “*hip eight weeks, the ribs might be slightly quicker*”. The fourth and final reference to time was given this included the potential for a problem in the future, for instance, P17 recognised that there could be ‘something’ wrong identified during recovery which, will, in the future prevent him doing his job, he identified this within a context of having a lot of injuries that needed to heal and that this took time and would occur in an order. Alternatively, P15 identified “*they [HCPs] made me aware that a year down the line things can still go wrong with the grafting*”.

Master plot 3 time point 1: Wanting or needing to look forward and accept; the birth of the quest

The basic plot line for the quest narrative was that I can embrace the present circumstances and use them, adapt and a valued future is possible. Slight variations in the quest narrative appeared to hinge on how acceptance varied between individuals. The ability to accept or embraced what had happened appeared varied according to specific strategies that enabled acceptance. This included (1) past experiences and knowing what to expect and there for accept, for instance, P14 and P17 identify past experiences and injuries which influence acceptance. For instance, P14 identifies accidences from epilepsy he states "*Not again. (Laughter) Yeah. Here we go again….I was not frightened at all. I know what to expect*." (2) identifying acceptance as not something there is a choice about. For instance, P10 identifies having acceptance to what he was being told without choice. (3) a reframing presentation of what had happened, essentially it is not how others see it, it is not being disabled because they are able. P14 who states "*other people might think of me as disabled. I think I'm extremely able. Yeah. It's a matter of attitude, isn't it?* " He goes on to illustrate an accepting nature of his attitude that makes everything useful or useable, akin to an adventure narrative. He considers how he embraces difficulties or challenges is stated "*Healing, physical healing takes longer when I'm just there depressed.... And, of course, having a good time is much more enjoyable. Yeah. There's always a silver lining. And if you can't find one, you can always create one....It's always been the same. I always was of the opinion, well, if life gives you lemons, make lemonade*". (4) the importance of working with what is possible at that moment in time. For instance P1 states "*I always just try and think about the positive of it. If I've hurt my knee, then I'll think okay, now I can do some more stuff with my upper body and just keep that in mind, in a physical sense, that's the way I sort of look at* it". (P1). (5) an attitude of problem solving. For instance, P12 identifies the importance of getting around problems he states "*I'm doing okay. I'm quite determined to get round it. And I can get about on my crutches quite good, in fact, I feel sometimes that I don't need these crutches*". (6) there was an attitude of holding expectations lightly, for instance, P24 identifies marking off what may not be possible in terms of function and the future. She states "*I am aware that that is maybe not possible, but I'm trying to sort of keep quite a neutral mindset, not worry one way or the other because I don't think that that's helpful*"

P20 and P18 identify the importance of changing as a result of what has happened illustrating the quest auto-mythology sub-type plot. P20 states *"" I just feel lucky to be alive you know and its completely changed my life, my attitude, you know I just want to look after myself more. I've lost, my weight is down, I've never been so… my weight has never been so low.* ". P18 identifies a transformation and quest auto mythology as part of his narrative because of the change that has occurred he states "*he has to 'learn to live with the injuries*', *'good clean living, eating healthily....get as mush rest and stick to their [health care professionals] advice*"

Stage 5 of the categorical analysis

During this stage critical consideration to the above descriptions were given and the final narratives were created.
